# Supplementary material for: Single Photon Kilohertz Frame Rate Imaging of Neural Activity
Source: Adv Sci (Weinh). 2022 Sep 6;9(31):2203018. doi: 10.1002/advs.202203018 (PMC9631062; doi:10.1002/advs.202203018)
Supplement: Supplementary file 1 — Supporting Information [file ADVS-9-2203018-s001.pdf]

Supplementary data

Type	emCCD	sCMOS		Binary SPAD
Model	Andor iXon Ultra 888	Zyla 4.2 PLUS	Hamamatsu ORCA-Flash4.0 V2/V3	SPCImager
GEVIs	QuasAr1&2[a]<br>SomArchon[b]<br>ASAP3[c]	SomArchon[a]<br>Voltron-JF$_{525}$-HTL[d]	QuasAr1&2[a]<br>paQuasAr3[e]<br>Voltron-JF$_{525}$-HTL[d]<br>Positron[f]	Voltron-JF$_{525}$-HTL (this manuscript)
Full-well capacity (e^-)*	80,000	30,000	30,000	1
Dynamic range	80,000:1	33,000:1	37,000:1	100,000:1
Peak Quantum efficiency	>95% (500-600nm)	82% @ 560nm	82% @ 560nm	35% (PDP) @ 450nm
Fill factor	100%	N/A	N/A	26.8%
Array size	1024 x 1024	2048 x 2048	2048 x 2048	320 x 240
Pixel size (µm)	13	6.5	6.5	8
Read noise (e^-)	<1 with EM gain	0.9	1.4	~0
Dark noise (dark count rate)	0.00025 Hz	0.10 Hz	0.05 Hz	25 Hz
Non-uniformity	N/A	< 0.1% PRNU	1% DRNU,0.5% PRNU	2% DRNU, 1% PRNU
Frame rate	26 fps (670 fps with 128 x 128 Crop Mode)	53 fps (26,041 fps from a 1024(h) x 8(v) ROI, 12 bit)	100 fps (9,329 fps from a 1024(h) x 8(v) ROI, 16 bit)	9,938.4 fps (binary frames)
Power dissipation	72 W	25 W (typical)	55 W	<1 W (sensor only)

Supplementary Table 1. Image sensor specification value comparison

The photo-response non uniformity (PRNU) is the spatial non-uniformity in sensitivity between pixels. The dark noise response non-uniformity (DRNU) is the spatial non-uniformity in noise between pixels. N/A, not applicable; PDP, photon detection probability (does not include fill factor). *The incident light is digitised by converting photons to electrons (e^-). Full well capacity is the number of electrons that can be stored within the well. Table adapted from Mai, et al., J. Soc. Inf. Disp. 26, 255–261 (2018). References: a, Hochbaum, D. R. et al., Nat. Methods 11, 825–833 (2014).; b, Piatkevich, K. D. et al., Nature 574, 413–417 (2019); c, Villette, V. et al., Cell 179, 1590–1608 (2019); d, Abdelfattah, A. S. et al., Science 365, 699–704 (2019); e, Adam, Y. et al., Nature 569, 413–417 (2019).; f, Kannan, M. et al., bioRxiv (2021).

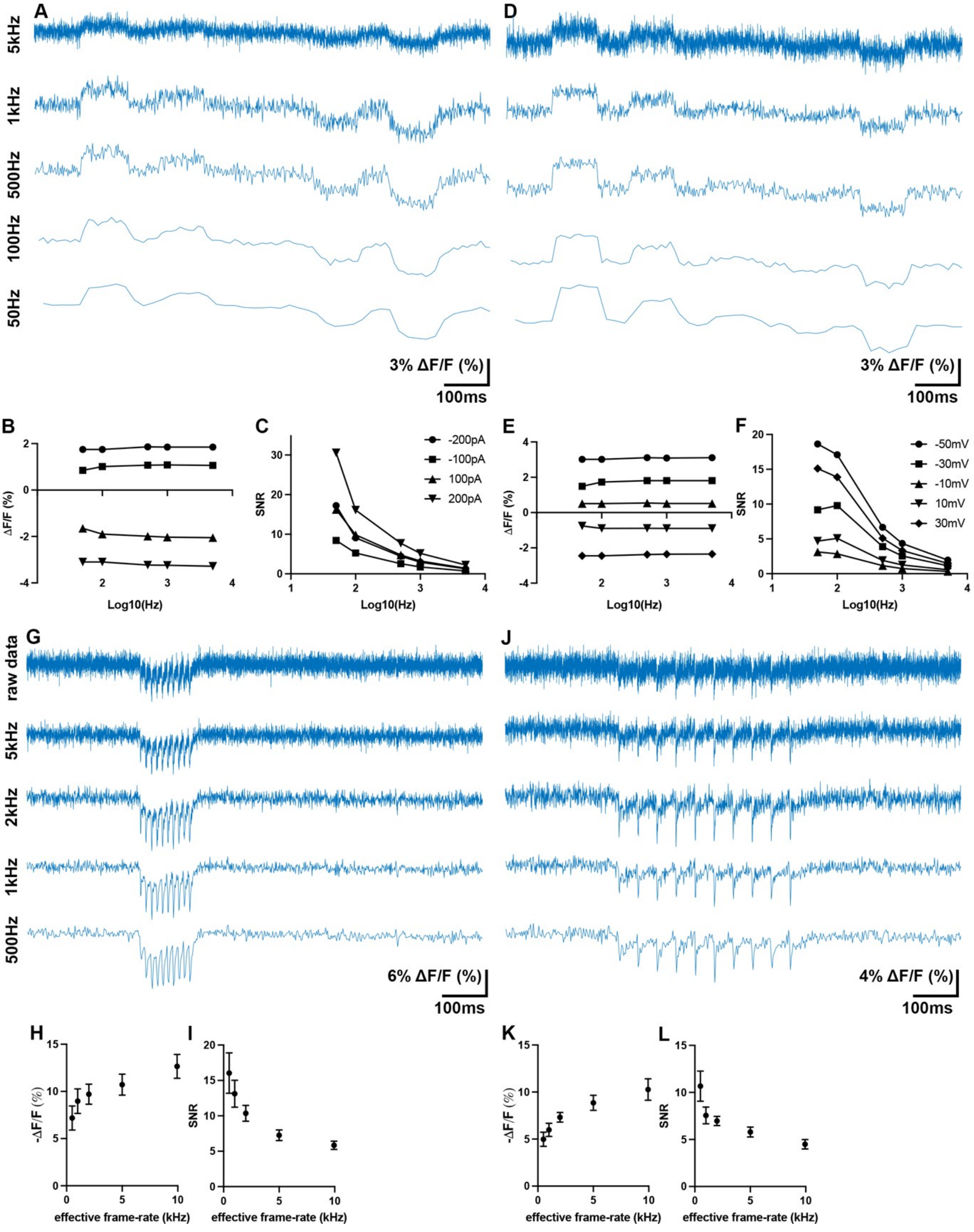

Supplementary figure 1. Effects of temporal binning on ΔF/F and SNR

(A, D) Representative optical traces of the same neuron subjected to current step (-200, -100, 100 and 200 pA) (A) and voltage step (-50, -30, -10, 10, 30 mV) (D) stimuli and temporally binned to achieve effective frame-rates of 50 Hz to 5 kHz. (B-C) Relationship between effective frame-rate and ΔF/F (B) and SNR (C) for each of the 4 current step stimuli in (A).

(E-F) Relationship between effective frame-rate and ΔF/F (E) and SNR (F) for each of the 4 voltage step stimuli in (D).

(G, J) Representative optical traces of the same neuron firing 10 action potentials at 100 Hz (G) and 25Hz (J) were temporally binned to achieve effective frame-rates of 500-5k Hz.

(H-I) Relationship between effective frame-rate and averaged ΔF/F (H) and SNR (I) of the 10 spikes in (G).

(K-L) Relationship between effective frame-rate and averaged ΔF/F (K) and SNR (L) of the 10 spikes in (J). (H, I, K, L), mean ± SEM.

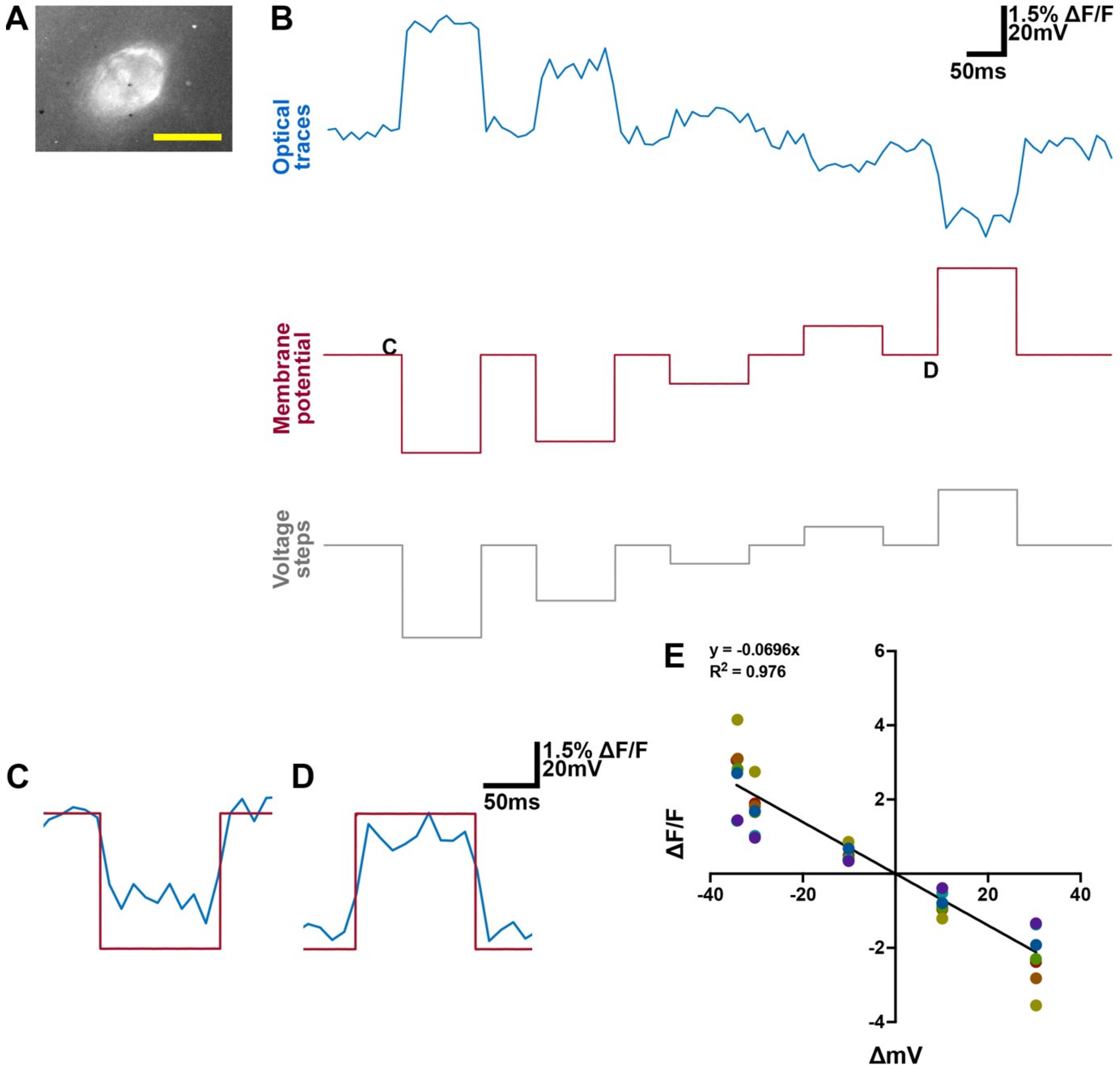

Supplementary figure 2. SPAD detection of responses to voltage step stimuli

(A) Image captured by the SPAD array of a patch-clamped neuron expressing Voltron-JF525-HTL, from which the traces in (B-D) was recorded. Scale bar for neuron image: 10µm.

(B) Single continuous traces from the neuron in (A) of simultaneous optical (blue) and electrical (red) recordings of membrane potential changes in response to voltage steps(grey). The optical traces were collected at a sampling at 9.9384 kHz, low pass filtered at 2 kHz and temporally binned at 100 Hz.

(C-D) Zoom-in of the segments with respective letters of the traces in panel B, with the optical (blue) and electrical (red) traces superimposed.

(E) The relationship between ΔF/F and changes in membrane potential (ΔmV) (n = 7 cells). The same cells with different current steps input were marked with the same colour. Simple linear regression was applied with intercepts set at x = 0 and y = 0, $p< 0.0001$.

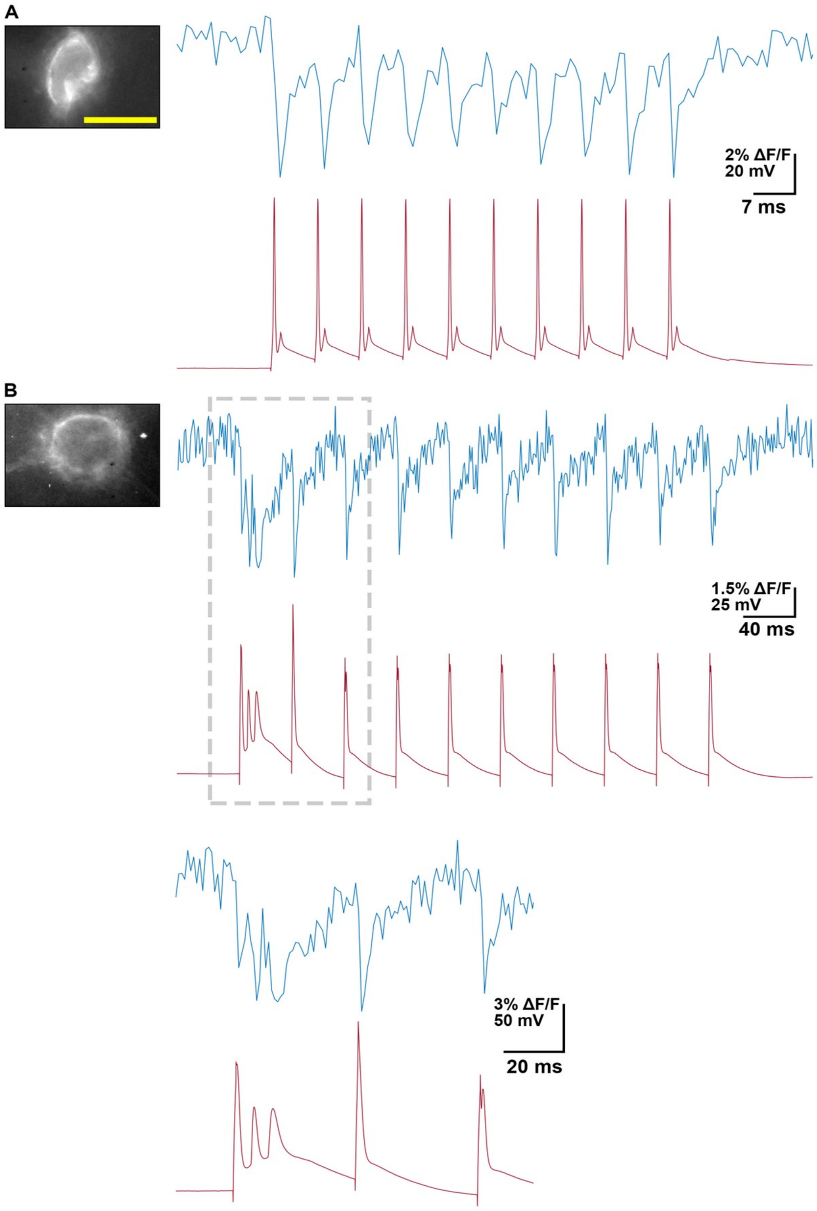

Supplementary figure 3. The SPAD image sensor can detect trains of action potential up to 150 Hz and complex spikes reported by Voltron-JF525-HTL.

(A) Single continuous traces of simultaneous optical (blue) and electrical (red) recordings of the neuron on the left firing 10 action potentials at a frequency close to 150 Hz.

(B) Single continuous traces of simultaneous optical (blue) and electrical (red) recordings of the neuron on the left showing complex spikes fired in response to one current pulse of 2 nA with 1 ms duration, showing SPAD can also record complex spikes reported by Voltron-JF525-HTL. The traces at the bottom show the traces in the segmented rectangle in (B) on an expanded time scale. Scale bar: 10µm.

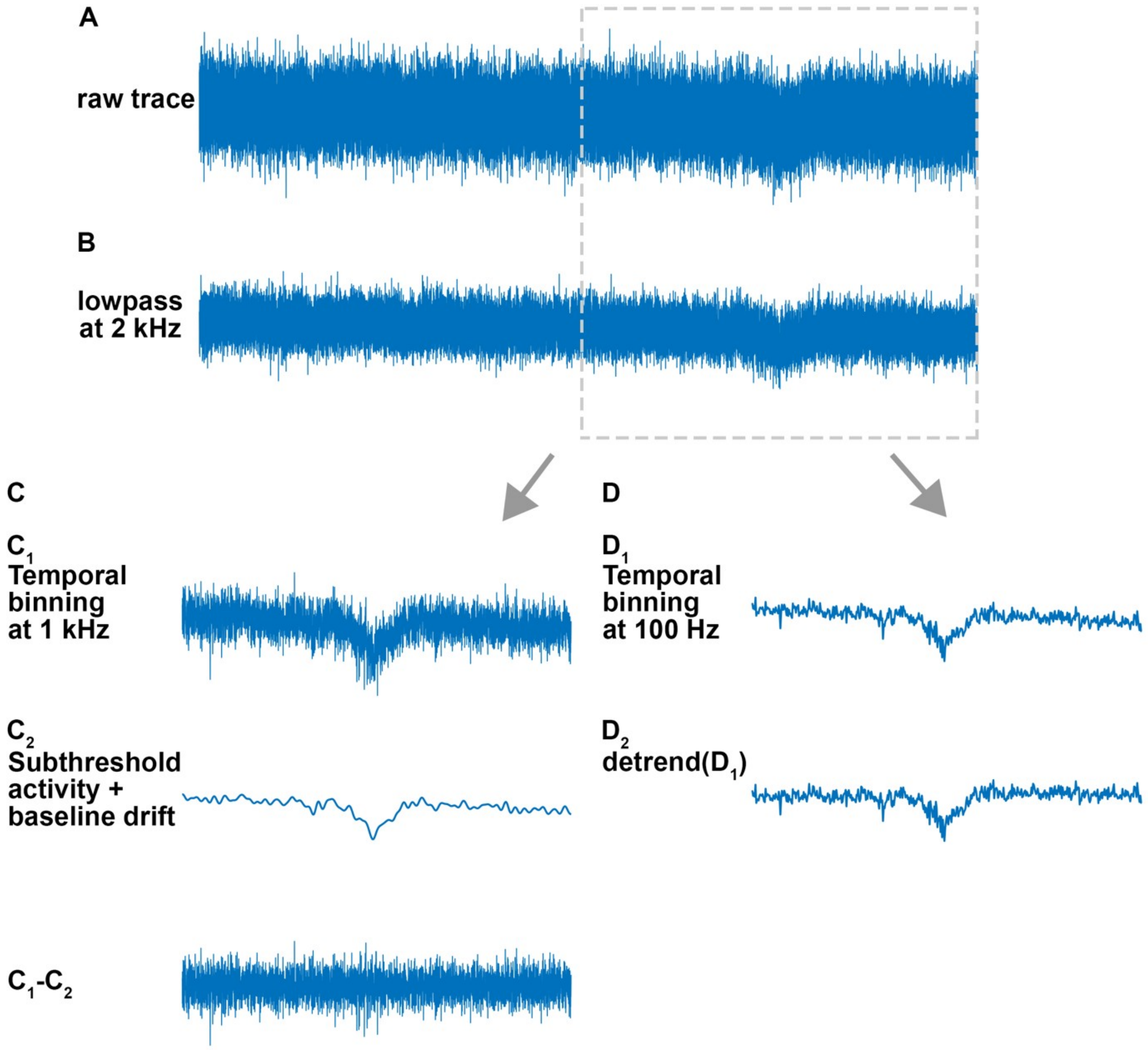

Supplementary figure 4. Analysis of sub-threshold and spike components of optical signals

(A-B) Raw 10-second duration optical trace recorded by the SPAD sensor and then low pass filtered at 2 kHz. The region included in the segmented rectangle was used as the example trace for processing steps in (C-D).

(C) The optical trace in (B) that was temporally binned to an effective frame rate of 1 kHz (C_1). Subthreshold activities and baseline-drifting due to photobleaching (C_2) were removed by low-pass filter at 10 Hz. Spike detection was then carried on the optical traces generated by C_1-C_2.

(D) Subthreshold activities were revealed by temporally binning the optical traces in (B) at 100 Hz (D_1) and removing the baseline-drifting due to photobleaching (D_2).

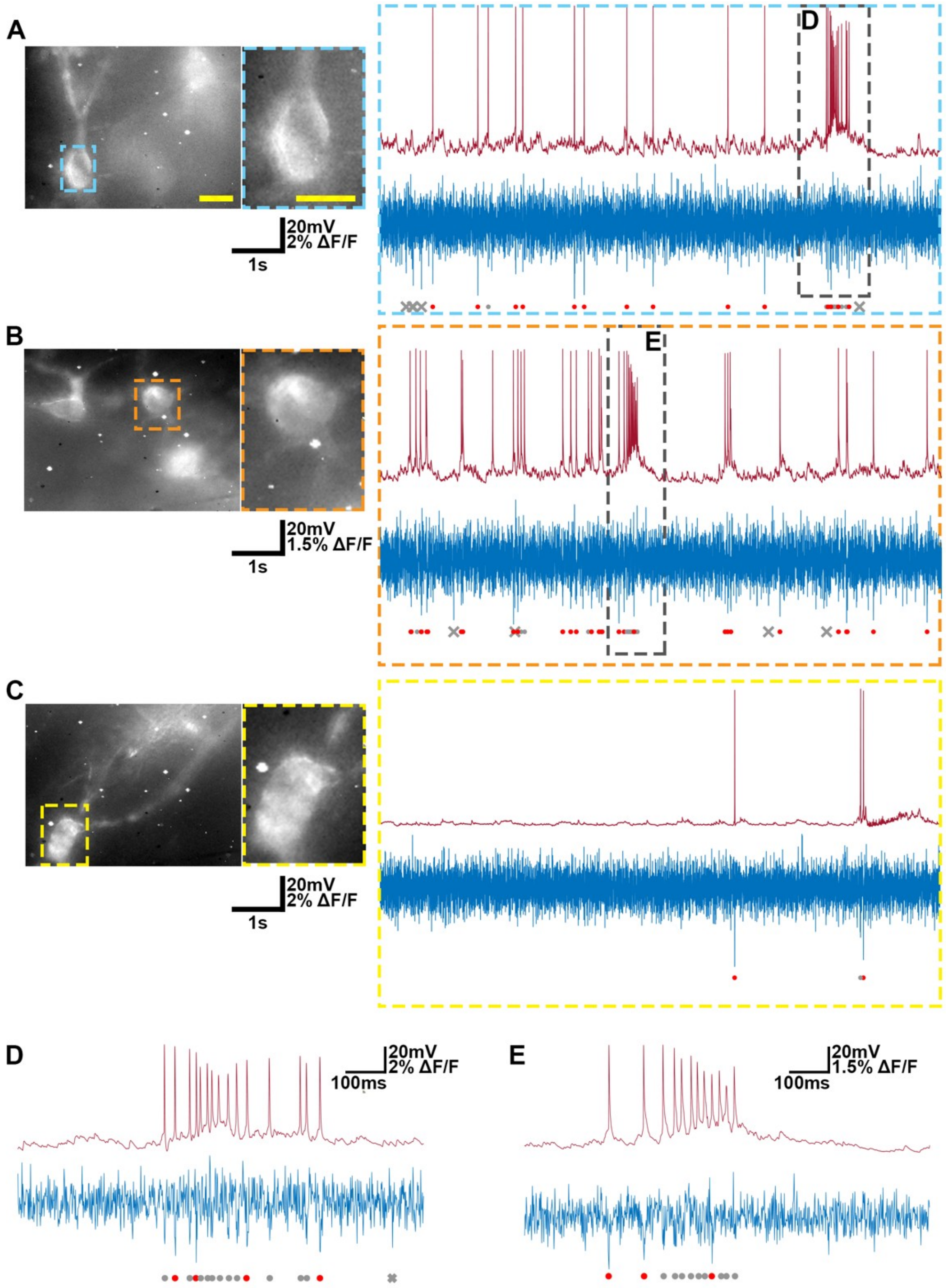

Supplementary figure 5. Detection of spikes during epileptiform activity

(A-C) Examples for three neurons of membrane potential recorded with patch-clamp (upper right), the corresponding optical trace baseline-subtracted and binned at 1 kHz as in Supplemental Figure 5 (lower right), and images of the field of view and region of interest for analysis (left). Correctly detected spikes are marked with a red dot, false positive spikes with a grey cross and false negative spikes with a grey dot. Scale bar: 10µm.

(D-E) Regions indicated in A and B on an expanded time base to illustrate signal and spike detection during activity bursts.

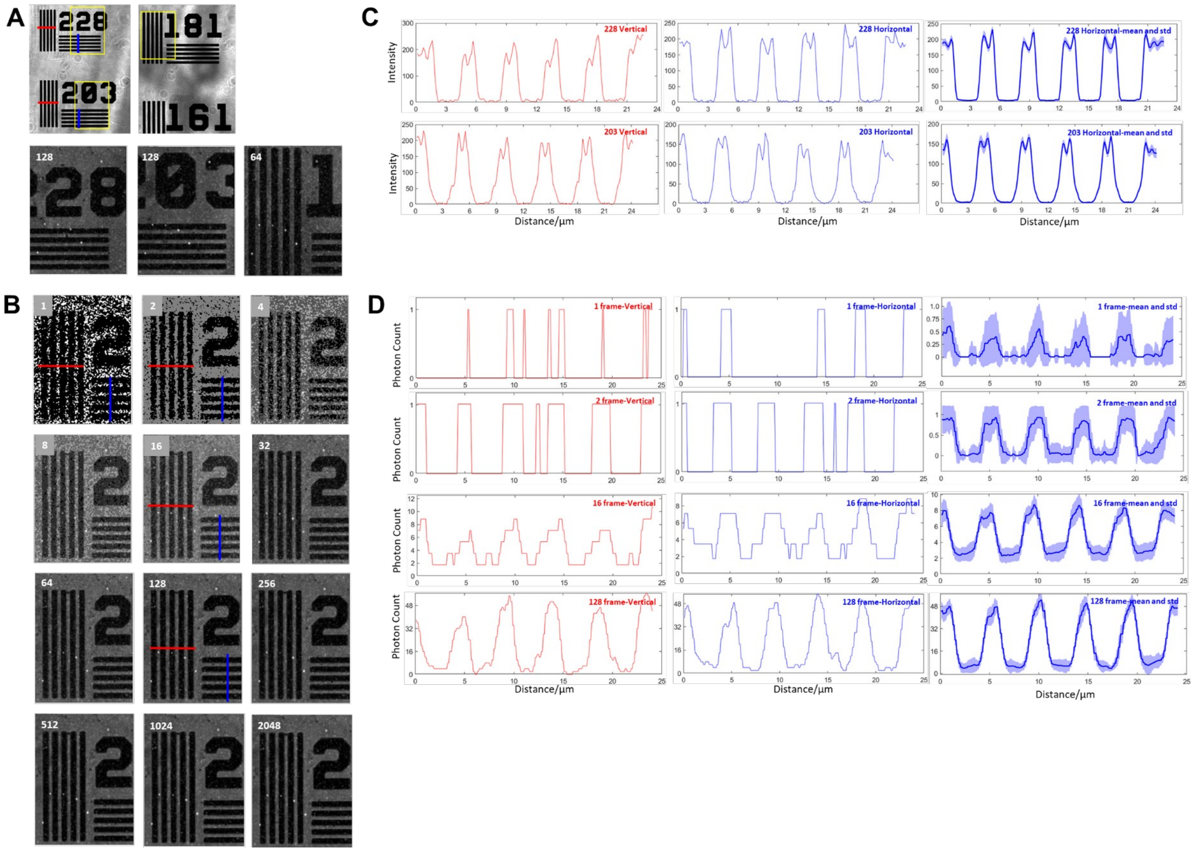

Supplementary figure 6. Resolution test of microscopy setup with SPAD camera.

(A) Images of NBS 1963A Resolution Test Targets taken by a digital CCD camera (Scientifica)(top) and corresponding images taken by the SPAD camera (bottom). The yellow boxes indicate the FOVs of the SPAD camera. The white number on the left corner of each SPAD image indicates the aggregated number of frames for that image. The number on the target card indicates the grid resolution, e.g. “228” means 228 cycles/mm, or 4.4 μm for each line pair.

(B) Live mode images taken by the SPAD camera with different aggregated frame numbers. The white number on the left corner of each SPAD image indicates the number of frames that aggregated to get that image.

(C) Image intensity profiles of the vertical and horizontal grids taken by the CCD camera (left and middle columns) and the mean intensity profile along 100 pixels for the horizontal grids (right column). Red and blue traces correspond to the vertical and horizontal grids indicated in (A). Thick blue traces and shaded areas in the right column indicate the mean ± SD of the intensity profile. The x-axis represents the real distance of the target; the y-axis represents the light intensity across the image.

(D) Example image intensity profiles for the vertical and horizontal grids taken by the SPAD camera (left and middle columns) and the mean intensity profile along 100 pixels for the horizontal grids (right column). 1, 2, 16 and 128 aggregated images are shown as examples. Red and blue traces are profiles of the vertical and horizontal grids indicated by the bars in (B). Thick blue traces and shaded areas in the right column indicate the mean ± SD of the intensity profile. The x-axis represents the real distance of the target; the y-axis represents the photon count obtained by the SPAD camera of the specified aggregation.

Funding, acknowledgements and copyright

We thank Robert Henderson and Ian Duguid for helpful discussions and support. The project was supported by funding from the Wellcome Trust (ISSF3 award IS3-R2.36 to IG and MFN, and Investigator Award 200855/Z/16/Z to MFN), the BBSRC EastBio doctoral training programme, and EPSRC (EP/S001638/1). For the purpose of open access, the author has applied a CC BY public copyright licence to any Author Accepted Manuscript version arising from this submission.

Data availability

Data is available from https://datashare.ed.ac.uk/handle/10283/4486.

Conflict of Interest

The authors have declared no competing interest.
